# Impact of Elastin-Derived Peptide VGVAPG on Matrix Metalloprotease-2 and -9 and the Tissue Inhibitor of Metalloproteinase-1, -2, -3 and -4 mRNA Expression in Mouse Cortical Glial Cells In Vitro

**DOI:** 10.1007/s12640-018-9935-x

**Published:** 2018-07-30

**Authors:** Konrad A. Szychowski, Anna K. Wójtowicz, Jan Gmiński

**Affiliations:** 1 Department of Public Health, Dietetics and Lifestyle Disorders, Faculty of Medicine, University of Information Technology and Management in Rzeszow, Sucharskiego 2, 35-225 Rzeszow, Poland; 2 Department of Animal Biotechnology, Faculty of Animal Sciences, University of Agriculture, Redzina 1B, 30-248 Krakow, Poland

**Keywords:** Elastin-derived peptides, VGVAPG, Glial cells, MMP-2, MMP-9, TIMPs

## Abstract

Degradation products of elastin, i.e. elastin-derived peptides (EDPs), are involved in various physiological and pathological processes. EDPs are detectable in cerebrospinal fluid in healthy people and in patients after ischemic stroke. However, to date, no studies concerning the role of EDP in the nervous system were conducted. Matrix metalloproteinases (MMPs) and tissue inhibitors of metalloproteinases (TIMPs) play important roles during the repair phases of cerebral ischemia, particularly during angiogenesis and reestablishment of cerebral blood flow. Therefore, the aim of this study was to investigate the impact of the specific elastin-derived peptide VGVAPG on *Mmp-2*, *-9* and *Timp-1*, *-2*, *-3* and *-4* mRNA expression in mouse cortical glial cells in vitro. Primary glial cells were maintained in DMEM/F12 without phenol red supplemented with 10% fetal bovine serum and the cells were exposed to 50 nM, 1 and 50 μM of the VGVAPG peptide. After 3 and 6 h of exposition to the peptide, expression of *Mmp-2*, *-9* and *Timp-1*, *-2*, *-3* and *-4* mRNA was measured. Moreover, siRNA gene knockdown, cytotoxicity and apoptosis measurement were included in our experiments, which showed that VGVAPG in a wide range of concentrations exhibited neither proapoptotic nor cytotoxic properties in mouse glial cells in vitro. The peptides enhanced mRNA expression of *Timp-2* and *Timp-3* genes in an elastin-binding protein (EBP)-dependent manner. However, changes in mRNA expression of *Mmp-2*, *Mmp-9* and *Timp-4* were partially EBP-dependent. The decrease in mRNA expression of *Timp-1* was EBP-independent. However, further studies underlying the VGVAPG peptide’s mechanism of action in the nervous system are necessary.

**Electronic supplementary material:**

The online version of this article (10.1007/s12640-018-9935-x) contains supplementary material, which is available to authorized users.

## Introduction

Elastin is an essential protein in mammalian organisms and provides elasticity to many connective tissues such as the major arteries, lung, cartilage, elastic ligaments and skin. Products of proteolytic degradation of elastin, namely elastin-derived peptides (EDPs), are involved in various physiological and pathological processes (Gmiński et al. 1992, 1993). EDPs are detectable in cerebrospinal fluid (CSF) in healthy people (6.3 ng/mL) and in patients after ischemic stroke (129.5–205.0 ng/mL) (Nicoloff et al. 2008; Tzvetanov et al. 2008), which suggests involvement of EDPs in pathological conditions and/or the regeneration process. In different physiological and pathophysiological conditions, such as inflammation or atherosclerosis, elastin is prone to proteolytic degradation which frees Val-Gly-Val-Ala-Pro-Gly (VGVAPG)-containing fragments (Lombard et al. 2006; O’Rourke 2007). The VGVAPG (498.58 molecular weight (MW)) hexapeptide is repeated multiple times in elastin molecules and binds to 67-kDa elastin-binding protein (EBP) with high affinity (Blood et al. 1988; Senior et al. 1984). EBP is a catalytically inactive form of beta-galactosidase produced by alternative splicing of the *GLB1* gene (Hinek et al. 1993; Skeie et al. 2012). To date, it has been demonstrated that VGVAPG induces diverse biological effects through EBP, depending on the research model. The VGVAPG peptide induced normal human cell proliferations such as fibroblast, monocyte and cancerous, e.g. human astrocytoma (Jung et al. 1998; Senior et al. 1984). Furthermore, the VGVAPG peptide exhibits strong chemotactic properties in the murine lung carcinoma cell line (M27) and facilitates the invasion of human melanoma cells (WM35 and HT168-M1) (Blood et al. 1988; Pocza et al. 2008). In addition to its chemotactic properties, it has been shown that EDPs or the VGVAPG peptide also upregulated the expression of different metalloproteinases (Floquet et al. 2004; Siemianowicz et al. 2010).

Matrix metalloproteinases (MMPs) are a family of zinc-dependent extracellular matrix-degrading enzymes involved in diverse homeostatic and pathological processes (Agrawal et al. 2008; Crocker et al. 2004). MMP-2 and MMP-9 (gelatinase A and B, respectively) are expressed within the central nervous system (CNS) and perform important normal and pathological functions during development and adulthood (Crocker et al. 2004; Yong et al. 2001). A number of papers show the emerging roles of MMP-2 and MMP-9 and their natural inhibitors, tissue inhibitors of metalloproteinases (TIMPs) in the regulation of astrocytic and neuronal cell death (Cunningham et al. 2005). In addition, MMPs and TIMPs are likely to play important roles during the repair phases of cerebral ischemia, particularly during angiogenesis and reestablishment of cerebral blood flow (Cunningham et al. 2005; Vanmeter et al. 2001; Wang et al. 2014). These processes will have important implications for therapies using MMP inhibitors in stroke (Cunningham et al. 2005). To date, it has been shown that the VGVAPG peptide in concentrations of 100 ng/mL ≈ 200.57 nM and 200 ng/mL ≈ 401.14 nM enhances angiogenesis by promoting endothelial cell migration and tubulogenesis through upregulation expression of mRNA of membrane-type matrix metalloprotease-1 (*MT1-MMP*) and *MMP-2* (Robinet 2005). A similar result was obtained by Ntayi et al. (2004), who showed that cell culture plates coated with 100.28 or 401.14 μM of VGVAPG caused an increase in the expression and activation of MMP-2 and MT1-MMP in two melanoma (M1Dor and M3Da) cell lines. Furthermore, it was shown that adding 200 μg/mL ≈ 401.14 μM of the VGVAPG peptide to the culture medium upregulated MMP-2, MT1-MMP and TIMP-2 mRNA expression and activity in the human fibrosarcoma (HT-1080) cell line and thus increased invasiveness of HT-1080 cells (Brassart et al. 1998; Donet et al. 2014).

Data concerning the VGVAPG peptide in CNS are very poor and limited to a few publications. So far, it has been demonstrated that 200 nM of the VGVAPG peptide can stimulate dendrite formations in mouse primary neuron culture (Chang et al. 2008). Furthermore, in human glioblastoma multiforme cell lines CB74, CB109 and CB191 and the rat astrocytoma cell line C6 exposed to 500 ng/mL ≈ 334.28 nM of the (VGVAPG)_3_ peptide, mRNA expression of *MMP-2* dramatically increased with very low stimulation of *MMP-9* (Coquerel et al. 2009). The authors linked this high expression of *MMP-2* mRNA with an increasing number of migrating cells. Even though EDPs have been detected in ageing brains and different pathologies of the CNS, no studies on EDPs’ role on normal glial cells have been conducted so far.

The aim of this study was to investigate the impact of specific elastin-derived peptide Val-Gly-Val-Ala-Pro-Gly (VGVAPG) on matrix metalloprotease-2 and -9 (*Mmp-2*, *-9*) and the tissue inhibitor of metalloproteinase-1, -2, -3 and -4 (*Timp-1*, *-2*, -*3* and *-4*) mRNA expression in mouse cortical glial cells in vitro.

## Materials and Methods

### Reagents

DMEM/F12 1:1 (16-405-CVR) without phenol red was purchased from Corning (Manassas, USA). Trypsin, streptomycin, penicillin, glycerol, CHAPS, HEPES, dithiothreitol (DTT), NaCl, EDTA, calcein AM, Hoechst 33342 and dimethyl sulfoxide (DMSO) were purchased from Sigma–Aldrich (St. Louis, MO, USA). The substrate for caspase-3 (235400) was purchased from Merck (Darmstadt, Germany). The cytotoxicity detection kit and FastStart Universal Probe Master (Rox) were purchased from ROCHE Applied Science (Mannheim, Germany). The *Glb1* gene siRNA (sc-61342) was purchased from Santa Cruz Biotechnology (Santa Cruz, CA, USA). The VGVAPG peptide was synthesised by LipoPharm.pl (Gdańsk, Poland). Charcoal/dextran-treated fetal bovine serum (FBS) was purchased from EURx (Gdańsk, Poland). The cDNA reverse transcription kit – High Capacity cDNA – Reverse Transcription Kit and the TaqMan® probes corresponding to specific genes encoding *Actb* (Mm00607939_s1), *Mmp-2* (Mm00439498_m1), *Mmp-9* (Mm00442991_m1), *Timp-1* (Mm01341361_m1), *Timp-2* (Mm00441825_m1), *Timp-3* (Mm00441826_m1) and *Timp-4* (Mm01184417_m1) were obtained from Life Technologies Applied Biosystems (Foster City, CA, USA). Stock solutions of the VGVAPG peptide were prepared in DMSO and were added to DMEM/F12 medium. The final concentration of DMSO in the culture medium was always 0.1%.

### Glial Cell Culture

The experiments were performed on mouse glial cells cell culture isolated from the fetuses (17/18 embryonal day) of pregnant female Swiss mice. Animals were anesthetised with CO_2_ vapour and killed by cervical dislocation. All procedures were approved by a Bioethics Commission (no. 46/2014, First Local Ethical Committee on Animal Testing at the Jagiellonian University in Krakow), as compliant with European Union law. After isolation and digestion process, cells were centrifuged and the pellet was suspended in DMEM/F12 1:1 without phenol red supplemented with 10% fetal bovine serum (FBS), 100 U/mL penicillin, 0.10 mg/mL streptomycin and 250 ng/mL amphotericin B with modifications of the previously described method (Blomstrand and Giaume 2006; Vitvitsky et al. 2006; Wang et al. 1998), [see different glial culture media and the techniques review in Saura (2007)]. The cells were seeded at a density of 20 × 10^6^ cells/75 cm^2^ in culture flasks. The cultures of the glial cells were maintained at 37 °C in atmosphere containing 5% CO_2_. In the logarithmic phase, after reaching 90% confluence, the cells were collected and frozen in liquid nitrogen. This procedure kills neurons in culture and leaves the glial cells, which allows to collect a large number of cell banks and to store cells for further research. Before the experiment, the cells were thawed and seeded in culture flasks and cultured for approximately a week to reach 80–90% confluence. Then, the cells were trypsinised with 0.25% trypsin/0.05% EDTA and passaged on to an experimental plate.

### Lactate Dehydrogenase Cytotoxicity Assay

An increase in the amount of dead or plasma membrane-damaged cells results in an increase in lactate dehydrogenase (LDH) release in the culture medium (Koh and Choi 1987). After 24 and 48 h of treating the cells with a medium containing 100 pM–100 μM of the VGVAPG peptide, 100 μL of the culture supernatants was collected and incubated in a reaction mixture from the kit according to the manufacturer’s protocol. After 30 min, the reaction was stopped by adding 1 N HCl and absorbance at a wavelength of 490 nm was measured using the FilterMax F5 Multi-Mode microplate reader (Molecular Devices, Corp., Sunnyvale, CA, USA).

### Caspase-3 Activity

Caspase-3 activity was used as a marker of cell apoptosis and was assessed according to Nicholson et al. (1995). Cultured glial cells were lysed using lysis buffer (50 mM HEPES, pH 7.4, 100 mM NaCl, 0.1% CHAPS, 1 mM EDTA, 10% glycerol and 10 mM DTT) in 10 °C for 10 min. After initial incubation, the lysates were incubated with caspase-3 substrate Ac-DEVD-pNA at 37 °C. Cells treated with 1 μM staurosporine were used as a positive control (result not shown). After 30 min, absorbance of the lysates at 405 nm was measured using a microplate reader (FilterMax F5 Multi-Mode microplate reader). The amount of colorimetric product was continuously monitored for 120 min.

### Hoechst 33342 and Calcein AM Staining

Hoechst 33342 and calcein AM staining were used to determine DNA fragmentation and metabolic activity and/or cell viability of cultured glial cells. Mouse primary glial cells were exposed to 1 μM of the VGVAPG peptide and the cells were cultured for an additional 24 h. After this period, glial cells were stained according to previously described method (Szychowski et al. 2015). The cells were washed with phosphate-buffered saline (PBS) and exposed to Hoechst 33342 and calcein AM diluted in DMEM/F12 1:1 and added to the glial cells culture at a final concentration of 10 and 4 μM, respectively. The cells were incubated for 15 min in an atmosphere of 5% and CO_2_ at 37 °C, washed one time in PBS and visualised by using a fluorescence microscope (LSM 700, ZEISS). Cell fluorescence in microphotographs was quantified by densitometry using ImageJ 1.50b software (National Institutes of Health, Bethesda, USA).

### siRNA Gene Silencing Procedure

*Glb1* siRNA was used to inhibit *Glb1* gene expression in mouse primary glial cells. siRNA was applied for 7 h at a final concentration of 50 nM in antibiotic-free medium containing the siRNA transfection reagent INTERFERin, according to previously described method (Szychowski et al. 2017). The effectiveness of *Glb1* mRNA silencing with the use of 50 nM specific siRNA was verified by measuring the mRNA levels. Cells were treated with 1 μM of the VGVAPG peptide and after 3 and 6 h, expression of genes encoding *Mmp-2*, *Mmp-9*, *Timp-1*, *Timp-2*, *Timp-3* and *Timp-4* was determined.

### Real-Time PCR Analysis of mRNAs Specific to Genes Encoding *Mmp-2*, *Mmp-9*, *Timp-1*, *Timp-2*, *Timp-3* and *Timp-4*

For the real-time PCR assay, glial cells were seeded on 12-well plates and initially cultured for 24 h. After 3 and 6 h of exposure to 50 nM and 1 and 50 μM of VGVAPG, samples of total RNA were extracted from glial cells according to the manufacturer’s protocol based on a previously described method (Szychowski et al. 2017). After siRNA transfection, the procedure was performed after 3 and 6 h of exposure for a concentration of 1 μM of VGVAPG. The RNA quality and quantity were determined spectrophotometrically at 260 nm and 280 nm (ND/1000 UV/Vis; Thermo Fisher NanoDrop, USA). Two-step real-time RT-PCR was conducted using the CFX Real Time System (BioRad, USA). The reverse transcription (RT) reaction was performed at a final volume of 20 μL with 180 ng of RNA (as a cDNA template) using the cDNA reverse transcription kit according to the manufacturer’s protocol. Products from the RT reaction were amplified using the FastStart Universal Probe Master (Rox) kit with TaqMan probes as primers for specific genes encoding *Actb*, *Mmp-2*, *Mmp-9*, *Timp-1*, *Timp-2*, *Timp-3* and *Timp-4* according to the manufacturer’s protocol. Amplification was carried out in a total volume of 20 μL containing 1.5 μL of the RT product, and β-actin was used as the reference gene.

### Statistical Analysis

The data are presented as the means ± SD of three independent experiments. Each treatment in experiment was repeated six times (*n* = 6) and measured in triplicate. The average of the triplicate samples was used for the statistical analyses. Statistical analysis was performed on the original results. Considering the different data from the measurement of absorbance, the results were presented as a percentage of controls (LDH and caspase-3 measurements). The data were analysed via one-way analysis of variance (ANOVA) followed by Tukey’s multiple comparison procedure in STATISTICA 10 software.

## Results

### Impact of VGVAPG on Cell Viability Measurements

#### LDH Cell Viability Assay

**Fig. 1:**
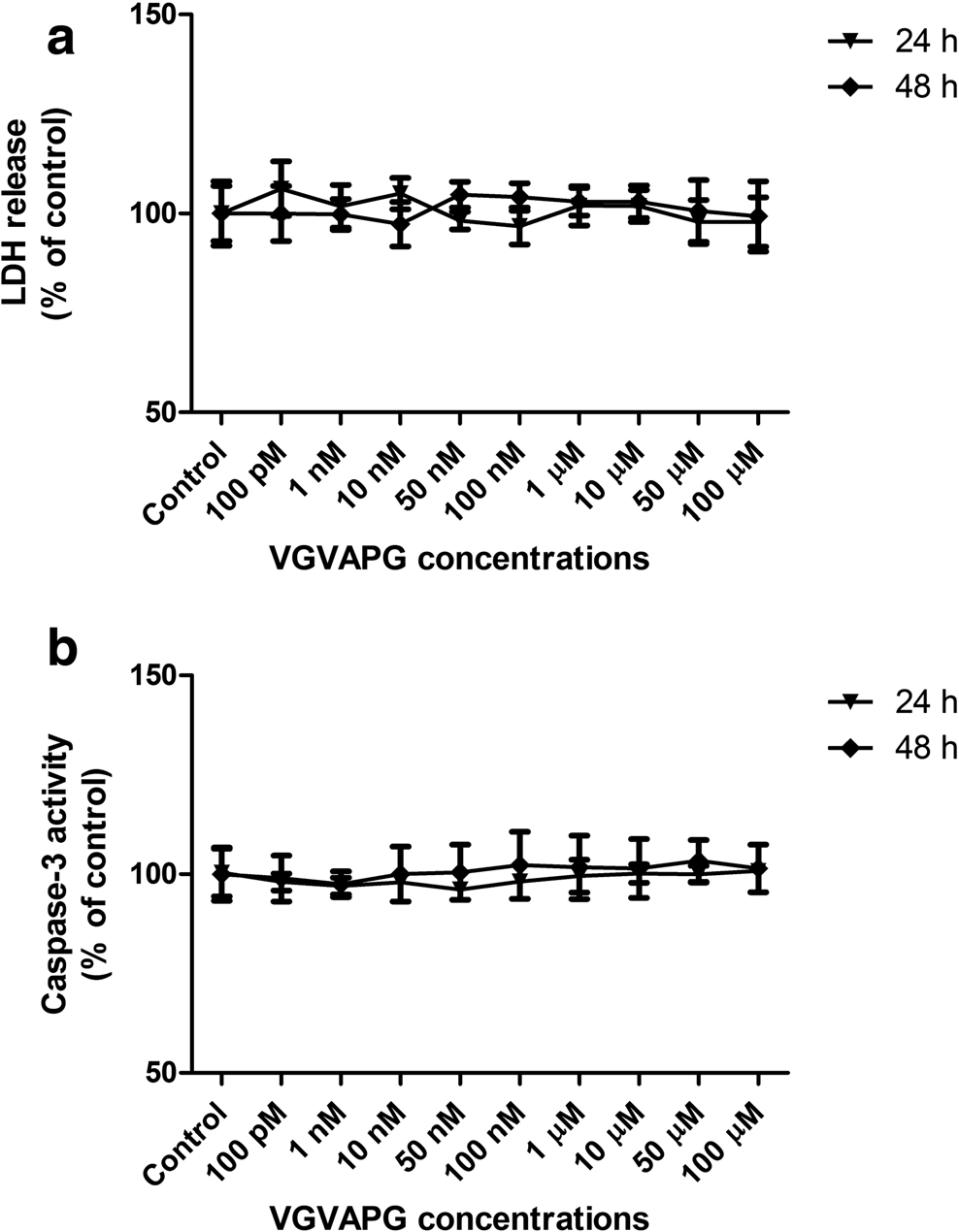
Effect of the VGVAPG peptide on LDH release (**a**) and caspase-3 activity (**b**) after 24 and 48 h in mouse primary glial cells in vitro. Data are expressed as means ± SD of three independent experiments, each of which consisted of eight replicates per treatment group

After 24 and 48 h of exposure of primary mouse glial cells to the studied VGVAPG peptide in concentrations ranging from 100 pM to 100 μM, we observed a lack of stimulation of LDH release (Fig. 1a).

#### Caspase-3 Activity

After 24 and 48 h of exposure of primary mouse glial cells to the studied VGVAPG peptide in concentrations ranging from 100 pM to 100 μM, we observed a lack of stimulation of caspase-3 activity (Fig. 1b).

### Cell Staining

**Fig. 2:**
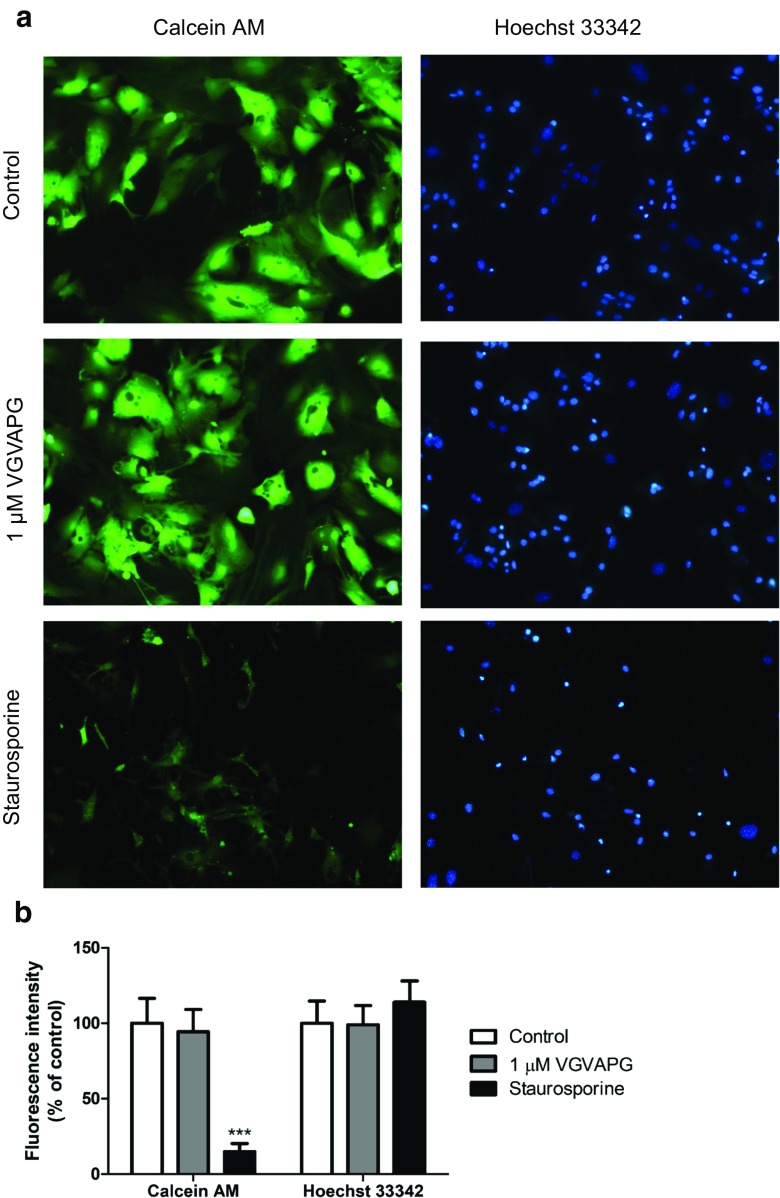
Effect of the VGVAPG peptide on Hoechst 33342 and calcein AM staining in cultured mouse glial cells in vitro 24 h post-treatment. **a** Control cells stained with calcein AM; control cells stained with Hoechst 33342; cells treated with 1 μM of the VGVAPG peptide and stained with calcein AM; cells treated with 1 μM of the VGVAPG peptide and stained with Hoechst 33342; cells treated with 1 μM of staurosporine and stained with calcein AM; cells treated with 1 μM of staurosporine and stained with Hoechst 33342. Cells with a light-coloured cytoplasm were identified as live cells. Cells with bright, fragmented nuclei containing condensed chromatin were identified as apoptotic. Photomicrographs are shown at × 200 magnification. **b** Cell fluorescence in microphotographs was quantified by densitometry in six replications by ImageJ 1.50b

Primary mouse glial cells were stained with Hoechst 33342 and calcein AM to determine the cell number and to assess their viability and/or metabolic activity. After 24 h of exposition of the cells to 1 μM of the VGVAPG peptide, no changes in the number of cells and their viability in comparison to the control cells were detected (Fig. 2).

### Impact of VGVAPG on mRNA Expression Levels in Mouse Cortical Glial Cells

#### Expression of *Mmp-2* and *Mmp-9* mRNA

**Fig. 3:**
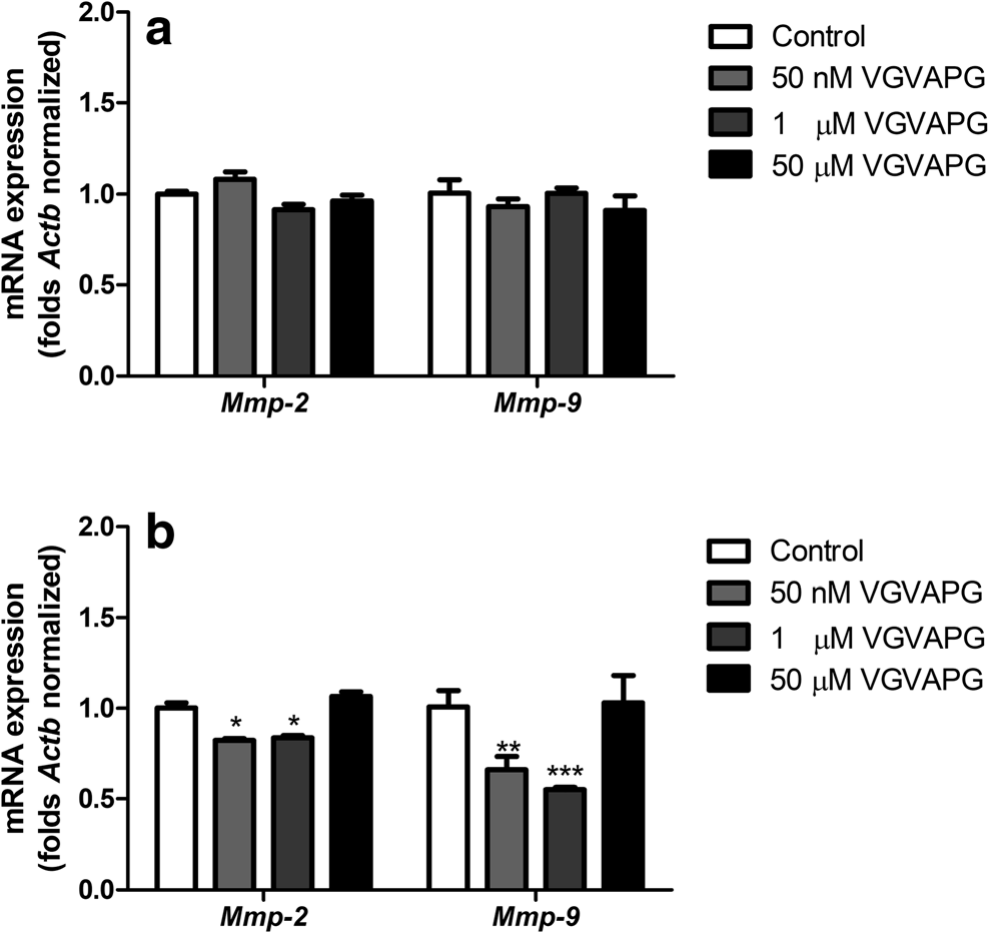
Effect of 50 nM, 1 and 50 μM of the VGVAPG peptide on mRNA expression of *Mmp-2* and *Mmp-9* after 3 h (**a**) and 6 h (**b**) of exposure. mRNA expression was normalised to *Actb*. Data are expressed as means ± SD of three independent experiments, each of which consisted of six replicates per treatment group; *P < 0.05; **P < 0.01; ***P < 0.001 vs. the vehicle control

Primary mouse glial cells were exposed to 50 nM, 1 and 50 μM of VGVAPG for 3 and 6 h. After 3 h of exposure to all VGVAPG peptide concentrations, we observed no change in *Mmp-2* or *Mmp-9* mRNA expression as compared to the control (Fig. 3a). However, after 6 h, there was a decrease in mRNA expression of *Mmp-2* and *Mmp-9* in cells exposed to 50 nM and 1 μM of VGVAPG as compared to the control (*Mmp-2* mRNA expression decreased by 17.7 and 16.3% respectively as compared to the control; *Mmp-9* mRNA expression decreased by 33.90 and 44.90% respectively as compared to the control) (Fig. 3b).

#### Expression of *Timp-1*, *Timp-2*, *Timp-3* and *Timp-4* mRNA

**Fig. 4:**
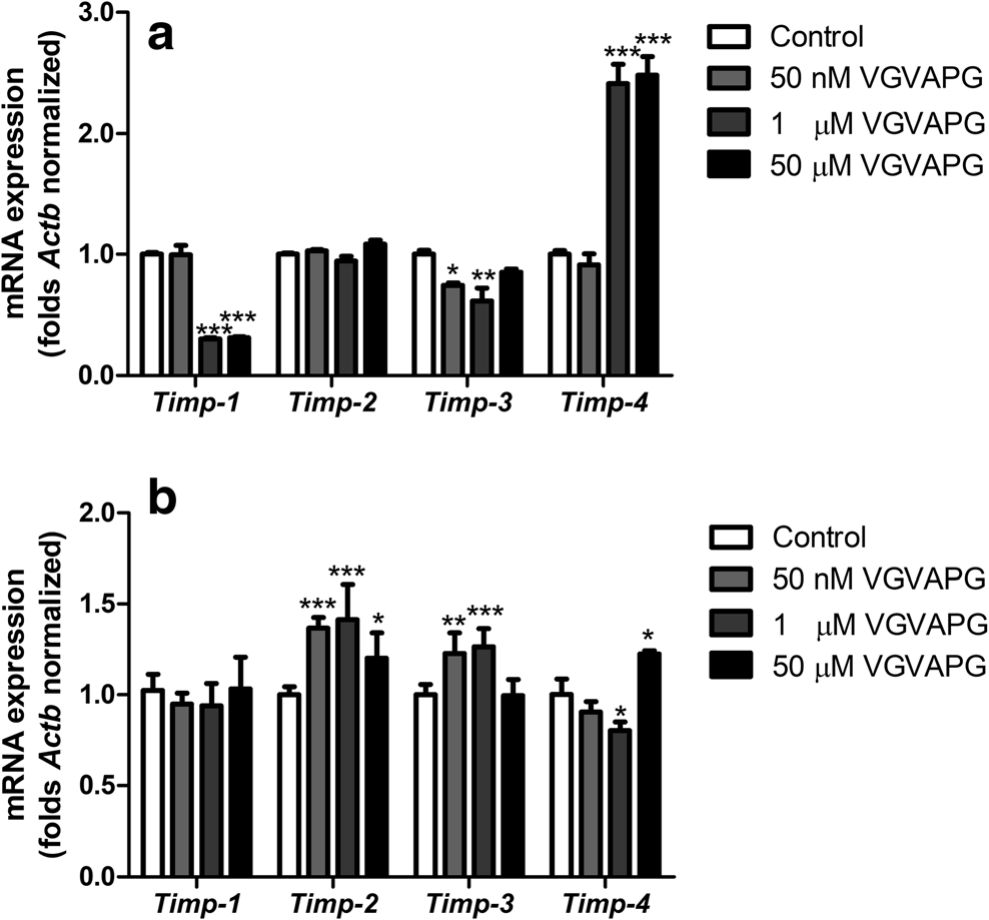
Effect of 50 nM, 1 and 50 μM of the VGVAPG peptide on mRNA expression of *Timp-1*, *Timp-2*, *Timp-3* and *Timp-4* after 3 h (**a**) and 6 h (**b**) of exposure. mRNA expression was normalised to *Actb*. Data are expressed as means ± SD of three independent experiments, each of which consisted of six replicates per treatment group; *P < 0.05; **P < 0.01; ***P < 0.001 vs. the vehicle control

After 3 h of exposure to 1 and 50 μM of VGVAPG, a decrease in *Timp-1* mRNA expression by 70.20 and 69.20% was observed as compared to the control. In the same time period, 50 nM and 1 μM of VGVAPG decreased *Timp-3* mRNA expression by 25.60 and 32.67%, respectively, as compared to the control. Moreover, in cells exposed to 1 and 50 μM of VGVAPG, expression of *Timp-4* mRNA increased by 140.66 and 147.00%, respectively, as compared to the control (Fig. 4a).

After 6 h of exposure to 1 μM of VGVAPG, no changes in *Timp-1* mRNA expression were observed. Moreover, for all of the studied concentrations of VGVAPG (50 nM and 1 and 50 μM), an increase in *Timp-2* mRNA expression by 36.80, 41.50 and 20.30%, respectively, was observed as compared to the control. Furthermore, concentrations of 50 nM and 1 μM of the VGVAPG peptide increased expression of *Timp-3* by 22.70 and 26.30%, respectively, as compared to the control. However, 1 μM of VGVAPG decreased *Timp-4* mRNA expression by 19.70% as compared to the control. Interestingly, 50 μM of VGVAPG increased *Timp-4* mRNA expression by 22.60% as compared to the control (Fig. 4b).

### Impact of VGVAPG on mRNA Expression Levels After Silencing the *Glb1* Gene by siRNA in Mouse Cortical Glial Cells

**Fig. 5:**
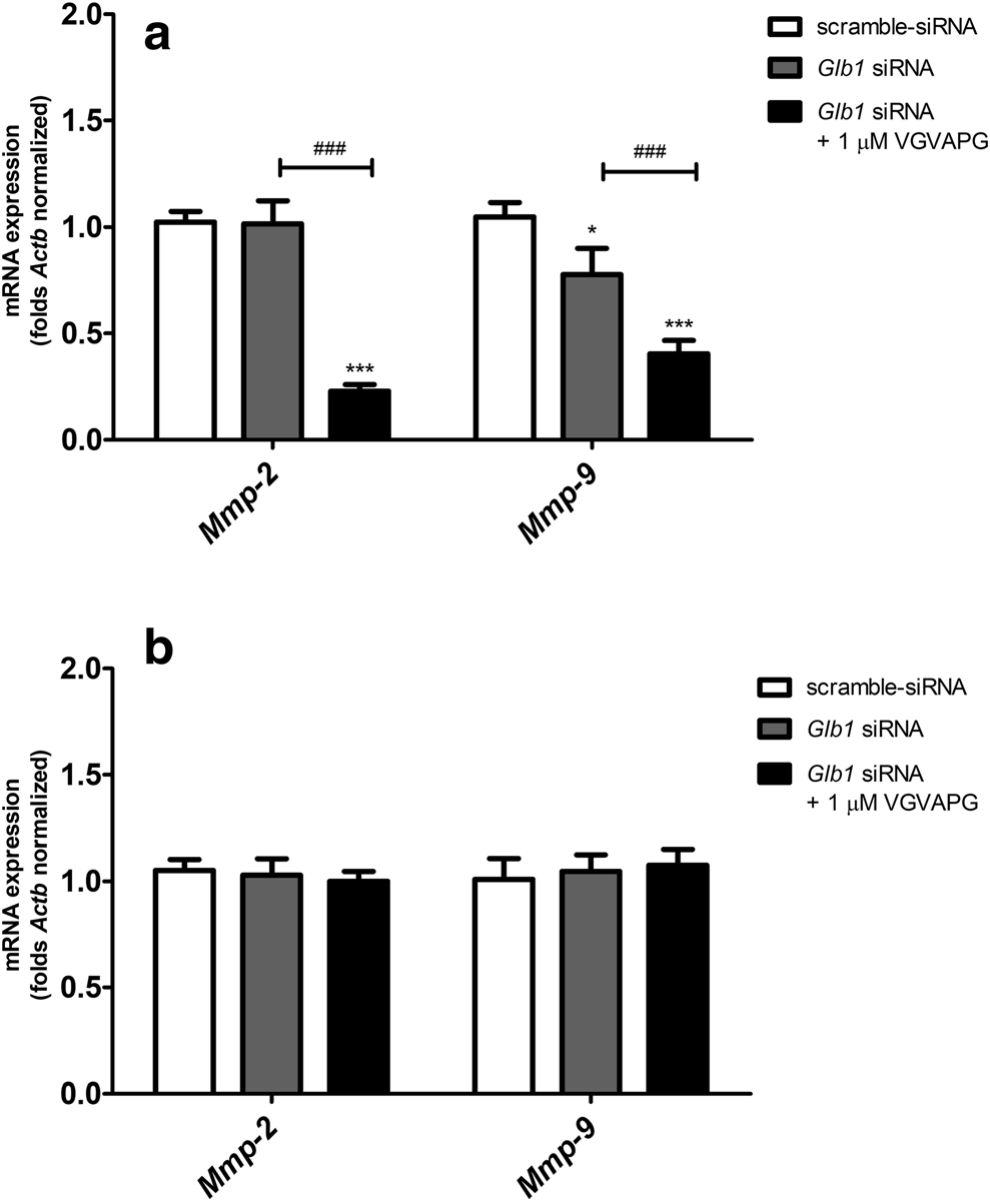
Effect of 1 μM of the VGVAPG peptide on mRNA expression of *Mmp-2* and *Mmp-9* after 3 h (**a**) and 6 h (**b**) of exposure. The experiment contains control with scramble-siRNA and control with *Glb1*-siRNA. The experiment was conducted 12 h after *Glb1* gene silencing by siRNA. mRNA expression was normalised to *Actb*. Data are expressed as means ± SD of three independent experiments, each of which consisted of six replicates per treatment group; *P < 0.05; ***P < 0.001 vs. scramble-siRNA control; ^**###**^*P* < 0.001 vs. *Glb1*-siRNA control

After the *Glb1* gene silencing procedure, 3 h of exposure to 1 μM of VGVAPG decreased mRNA expression of *Mmp-2* and *Mmp-9* (decreased by 77.00 and 37.40%, respectively) as compared to the *Glb1* siRNA control. However, the use of *Glb1* siRNA alone caused a decrease in the expression of *Mmp-9* mRNA as compared to the cells with scramble siRNA (decrease by 27.10%, as compared to the scramble control) (Fig. 5a). After 6 h of exposure to 1 μM of VGVAPG, expression of *Mmp-2* and *Mmp-9* genes did not change as compared to scramble-siRNA and *Glb1*-siRNA controls (Fig. 5b).

**Fig. 6:**
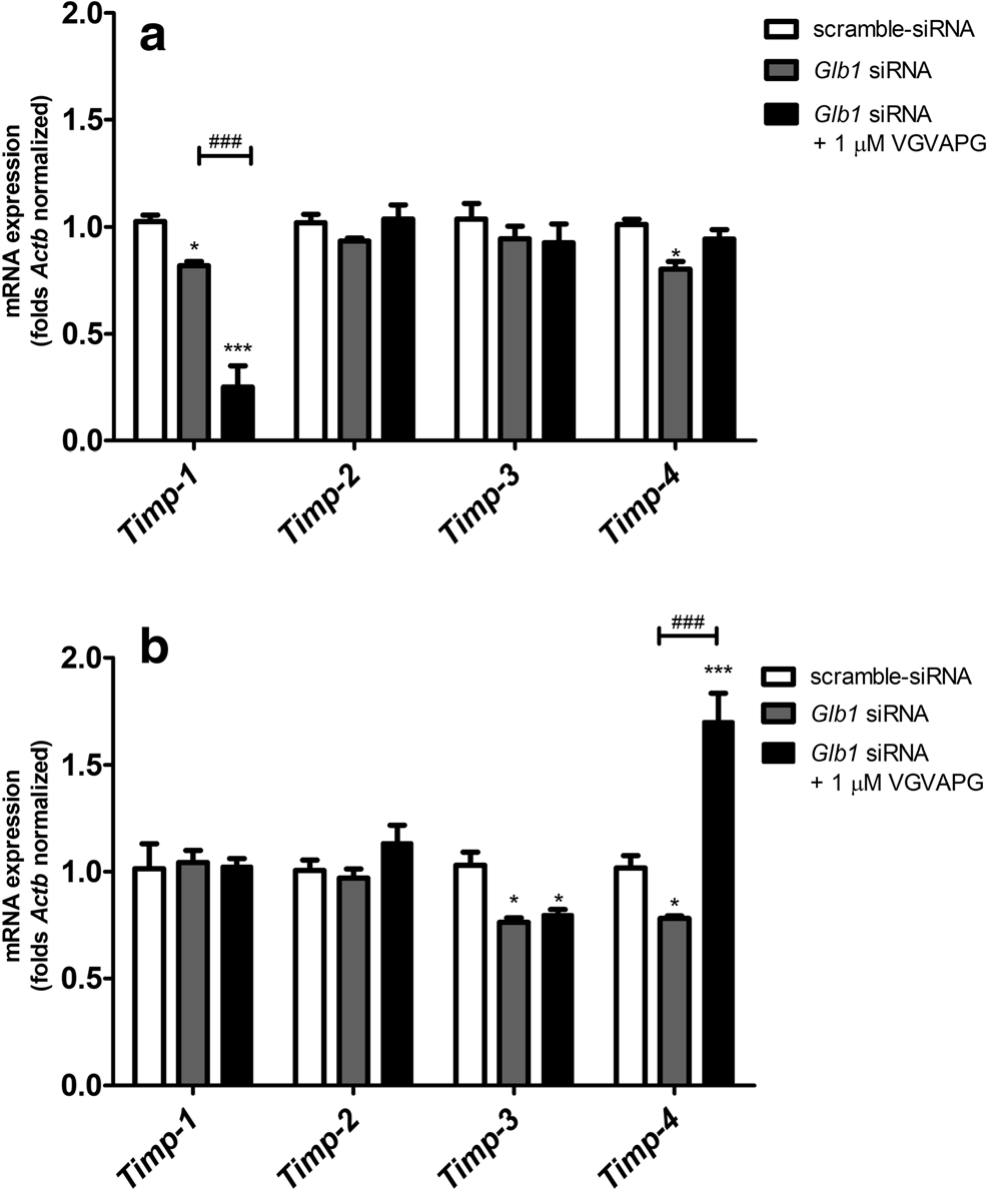
Effect of 1 μM of the VGVAPG peptide on mRNA expression of *Timp-1*, *Timp-2*, *Timp-3* and *Timp-4* after 3 h (**a**) and 6 h (**b**) of exposure. The experiment contains control with scramble-siRNA and control with *Glb1*-siRNA. The experiment was conducted 12 h after *Glb1* gene silencing by siRNA. mRNA expression was normalised to *Actb*. Data are expressed as means ± SD of three independent experiments, each of which consisted of six replicates per treatment group; *P < 0.05; ***P < 0.001 vs. scramble-siRNA control; ^**###**^*P* < 0.001 vs. *Glb1*-siRNA control

After the *Glb1* gene silencing procedure, 3 h of exposure to 1 μM of VGVAPG decreased mRNA expression of *Timp-1* by 56.80%, as compared to the *Glb1*-siRNA control. However, the use of *Glb1* siRNA alone caused a decrease in the expression of *Timp-1* and *Timp-4* mRNA as compared to the cells with scramble siRNA (decrease by 20.80 and 20.90%, respectively, as compared to the scramble control) (Fig. 6a). After 6 h of exposure to 1 μM of VGVAPG, a *Timp-4* mRNA increase by 91.90% as compared to the *Glb1*-siRNA control was observed. However, the use of *Glb1* siRNA alone caused a decrease in the expression of Timp-4 mRNA as compared to the cells with scramble siRNA (decrease by 23.60%, as compared to the scramble control). The expression of *Timp-3* mRNA decreased by 26.80% as compared to scramble-siRNA-treated cells. The expression of *Timp-1* and *Timp-2* mRNA genes did not change as compared to the scramble-siRNA control (Fig. 6b).

**Fig. 7:**
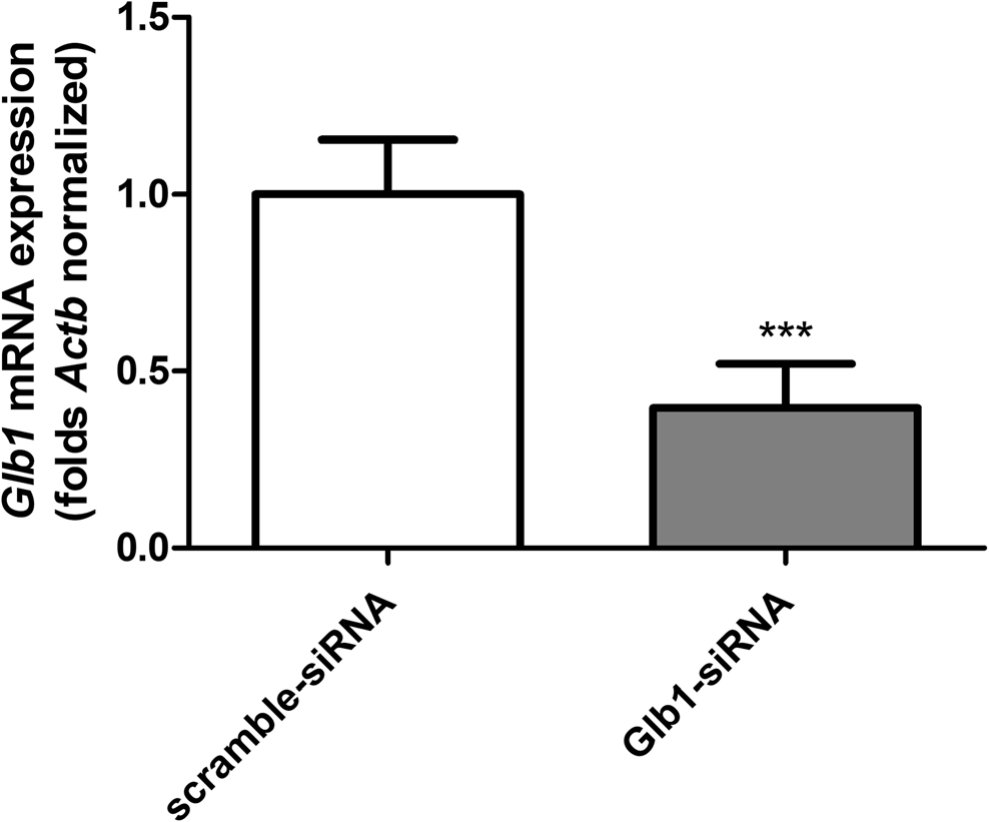
Effect of *Glb1* gene silencing by siRNA on *Glb1* gene mRNA expression. mRNA expression was normalised to *Actb*. Data are expressed as means ± SD of three independent experiments, each of which consisted of six replicates per treatment group; ***P < 0.001 vs. the vehicle control

siRNA transfection efficiencies were established by qPCR method. Knockdown of *Glb1* gene was estimated at 39% of the vehicle control mRNA (Fig. 7).

## Discussion

Our study investigated the elastin-derived peptide, VGVAPG, mechanism of action in mouse primary glial cells in vitro. It is known that peptides liberated from a degraded extracellular matrix may interact with different receptors on the cell surface which results in the activation of intracellular signalling pathways which can lead to cellular events as diverse as cell adhesion, migration, proliferation, protein synthesis or apoptosis (Maquart et al. 2004). Due to a lack of data concerning the VGVAPG peptide mechanism of action in CNS, first we investigated its cytotoxic or proapoptotic potential. Our data showed that the VGVAPG peptide was not cytotoxic and did not activate apoptotic cell death in a broad spectrum of concentrations (100 pM to 100 μM) and in all of the studied time intervals (24 and 48 h) in mouse primary glial cells in vitro. A lack of apoptosis is important because the number of EDPs highly increases after a stroke and is accompanied by an inflammation process that can finally lead to cell death (Jin et al. 2010; Nicoloff et al. 2008; Tzvetanov et al. 2008). Moreover, in cells stained with Hoechst 33342 and calcein AM dyes, we observed no changes in the cell morphology and cell viability after 24 h of exposition of primary glial cells in vitro to 1 μM of the VGVAPG peptide.

Our studies are the first to demonstrate that after 3 h of exposure to 50 nM, 1 and 50 μM of the VGVAPG peptide, mRNA expression of *Mmp-2* and *Mmp-9* did not change. After 6 h of exposure to 50 nM and 1 μM of the VGVAPG peptide, *Mmp-2* and *Mmp-9* mRNA expression decreased as compared to the control level. Interestingly, mRNA expression of both of the studied genes did not change in a concentration of 50 μM of VGVAPG. There are no data concerning *MMP-2* and *MMP-9* mRNA expression after stimulation of the VGVAPG peptide in normal cells derived from mouse or human CNS. The available data concerning cancer cells derived from the nervous system show that 100 ng/mL ≈ 66.86 nM of the (VGVAPG)_3_ peptide strongly upregulated *MMP-2* mRNA expression in the rat astrocytoma cell line C6 and human gliomas, such as CB74, CB109 and CB191 (Coquerel et al. 2009). In the same paper, the authors showed a very weak *MMP-9* mRNA expression in two of the four studied gliomas. To date, Mmp-2 and Mmp-9 mRNA and protein upregulation by the VGVAPG peptide are well documented in different cell culture models, but mainly in cancerous ones. The discrepancy between our results and mentioned data could be the result of normal and cancer cells differences. The possible mechanism of the decrease in MMP-2 and MMP-9 in glial cells could be connected with nitric oxide (ON) pathways. There has been shown a link between NO and *MMP-2* and *MMP-9* mRNA expression. Such interaction was previously detected in humane endothelial cells in which NO caused the decrease in expression of both *MMP-2* and *MMP-9* mRNA (Chen and Wang 2004; Phillips and Birnby 2004). Moreover, a similar association has been shown in rat primary astrocytes, where downregulation of *Mmp-9* mRNA levels was caused by NO (Shin et al. 2007). It is known that the VGVAPG peptide has high affinity to EBP (Devy et al. 2010; Robinet 2005; Senior et al. 1984). Therefore, we decided to use specific siRNA targeting in the *Glb1* gene which is alternatively spliced in EBP. In glial cells with *Glb1* gene knockdown, 3 h of exposure to VGVAPG decreased *Mmp-2* and *Mmp-9* mRNA expression. The obtained data suggest that *Mmp-2* and *Mmp-9* mRNA expression can be partially dependent on EBP activation.

After secretion from producing cells, MMP activity can be regulated by TIMPs (Kurzepa et al. 2014). TIMP-1 inhibits most of the MMPs with the highest affinity to MMP-9, but TIMP-2 and TIMP-4 have the highest affinity to MMP-2 (Brew et al. 2000; Trojanek 2015). TIMP-3 is different than the other TIMPs, as it is insoluble, attached to the extracellular matrix (ECM) and inhibits disintegrin and metalloproteinase (ADAMs) with high affinity (Crocker et al. 2004). However, TIMPs also perform other physiological functions. TIMP-1 and TIMP-2 are responsible for such functions as induction of erythropoiesis, mitogenesis and apoptosis (Docherty et al. 1985; Guedez et al. 1998b; Stetler-Stevenson et al. 1992). Moreover, astrocytic Timp-1 promotes oligodendrocyte differentiation and enhances myelination in CNS (Moore et al. 2011). TIMP-2 inhibits endothelial cell growth induced by fibroblast growth factor (Ahonen et al. 2003; Lipka and Boratyński 2008). TIMP-3 also shows proapoptotic properties due to stabilisation of the Fas receptor (FasR), while TIMP-1 and TIMP-2 can also exhibit anti-apoptotic properties (Cawston and Mercer 1986; Guedez et al. 1998a). Moreover, an increase in mRNA expression of both *Timp-2* and *Timp-3* has been observed in places of increased neurogenesis, cell maturation and migration (Jaworski and Fager 2000; Vaillant et al. 1999). To date, the role of TIMP-4 continues to be poorly understood, although it has been shown that TIMP-4 activated apoptosis of fibroblasts from cardiac muscle and inhibited migration of cells capable of forming capillaries, while there was no impact on the process of proliferation and angiogenesis (Koskivirta et al. 2006).

Our data are the first to show that after 3 h of exposure to 1 and 50 μM of the VGVAPG peptide, *Timp-1* mRNA expression in glial cells was significantly decreased. However, after 6 h of exposure to 1 and 50 μM of the VGVAPG peptide, the *Timp-1* mRNA expression pattern did not change as compared to the control. Silencing of the *Glb1* gene did not change the *Timp-1* mRNA expression pattern caused by 1 μM of the VGVAPG peptide. Such data suggest that in *Timp-1*, mRNA expression is regulated by an EBP-independent mechanism. To date, it has been shown that after birth in the rat brain, *Timp-1* mRNA expression significantly decreased (Fager and Jaworski 2000). Similarly, downregulation of Timp-1 mRNA and protein expression was observed in Sprague Dawley rats with ischemic-reperfusion (Ma et al. 2016). In patients with end-stage neurological disease, chronic activation of astrocytes by IL-1β caused downregulation of TIMP-1 mRNA and protein expression in CSF and brain tissues (Gardner and Ghorpade 2003).

In our experiments, *Timp-2* and *Timp-3* mRNA expression significantly increased after 6 h of exposure to 50 nM and 1 μM of the VGVAPG peptide. However, knockout of the *Glb1* gene abolished the effects of 1 μM of VGVAPG peptide action. Our data would suggest that activation of *Timp-2* and *Timp-3* mRNA expression is mainly EBP-dependent. To date, it has been reported that increased expression of TIMP-3 enhances Fas-mediated cell death through stabilisation of the FasR as well as by preventing shedding of FasL from the extracellular matrix (Ahonen et al. 2003; Mitsiades et al. 1998). Furthermore, it has also been demonstrated that integrin α3β1 as a TIMP-2 receptor results in inhibition of fibroblast growth factor (FGF) or VEGF-induced angiogenesis by effectively blocking the association of HSP60 and SHP-1 to this receptor (Seo et al. 2003). High mRNA expression of *Timp-2* and *Timp-3* was detected in zones of neurogenesis in rat brains and these proteins are considered important in cellular proliferation and/or differentiation (Jaworski and Fager 2000; Vaillant et al. 1999). Furthermore, enhanced *TIMP-3* mRNA expression was found in normal human proliferating keratinocytes during the wound healing process (Vaalamo et al. 1999). It has also been shown that TIMP-3 possesses growth factor-like properties and its presence is required for the growth of oligodendrocytes (Gomez et al. 1997; Oh et al. 1999).

After 3 h of exposure to 1 and 50 μM of VGVAPG peptide, *Timp-4* mRNA expression significantly increased; however, after 6 h of exposure to 50 nM and 1 μM of VGVAPG peptide, *Timp-4* mRNA expression decreased. Silencing the *Glb1* gene protected the cells against changes caused by 1 μM of VGVAPG peptide in *Timp-4* mRNA expression after 3 h; however, after 6 h, mRNA expression of *Timp-4* significantly increased. Our data suggest that *Timp-4* mRNA expression can be partially EDP-dependent. TIMP-4 has been poorly studied and data concerning its expression and function are limited. To date, it has been shown that TIMP-4 expression increases during damage of blood vessels and is correlated with a developing inflammation (Koskivirta et al. 2006). Moreover, upregulation of TIMP-4 in response to inflammation in CSF from patients with tropical spastic paraparesis or myelopathy associated with T cell lymphotropic virus was observed (Kettlun et al. 2003). Research conducted on human gliomas has shown that TIMP-4 substantially reduced glioma’s invasive capacity, although without decreased cell viability and proliferation (Groft et al. 2001). Currently, it is believed that TIMP-4 mRNA and protein upregulation appear in a pathological inflammation where extracellular matrix remodeling is usually induced and in the wound repair process (Koskivirta et al. 2006; Vaalamo et al. 1999).

Interestingly, Fager and Jaworski (2000) studied mRNA expression of *Timp-1*, *Timp-2*, *Timp-3* and *Timp-4* during rat central nervous system development. They showed that in whole-brain extracts, *Timp-1* mRNA slowly disappeared in favour of gradually increasing *Timp-4.* Furthermore, *Timp-2* and *Timp-3* mRNA slowly grew throughout the rat’s entire lifespan. The whole *Timp* mRNA expression pattern obtained in our study is consistent with that previously found by Fager and Jaworski (2000) during development of CNS.

## Conclusion

Our study is the first to have investigated the VGVAPG mechanism of action in mouse primary glial cells in vitro. Our experiments show that VGVAPG in a wide range of concentrations exhibits neither cytotoxic nor proapoptotic properties in mouse glial cells. Our research shows that an increase in mRNA expression of *Timp-2* and *Timp-3* genes in mouse glial cells in vitro is EBP-dependent. Nevertheless, changes in mRNA expression of *Mmp-2*, *Mmp-9* and *Timp-4* genes in mouse glial cells in vitro can be partially EBP-dependent. Decreased mRNA expression of *Timp-1* is probably EBP-independent. Our data suggest that VGVAPG peptides sensitise mouse glial cells in vitro to apoptotic or pro-inflammatory signals from the brain microenvironment. However, we cannot exclude that the increasing expression of *Timp-2*, *Timp-3* and *Timp-4* mRNA also facilitates brain repair after a stroke by increasing cell proliferation and/or differentiation. Our research is pioneering in this research field and has a preliminary character; further studies underlying VGVAPG peptide mechanisms of action in the nervous system are thus necessary.

## Electronic Supplementary Material

ESM 1 (PPTX 4022 kb)

## Abbreviations

CNS: Central nervous system
CSF: Cerebrospinal fluid
DMSO: Dimethyl sulfoxide
EDPs: Elastin-derived peptides
FasR: Fas receptor
FBS: Fetal bovine serum
LDH: Lactate dehydrogenase
PBS: Phosphate-buffered saline
ROS: Reactive oxygen species
MMP: Matrix metalloprotease
TIMP: Tissue inhibitor of metalloproteinase
VGVAPG: Val-Gly-Val-Ala-Pro-Gly
siRNA: Small interfering RNA
EBP: Elastin-binding protein

## References

[CR1] Agrawal SM, Lau L, Yong VW (2008). MMPs in the central nervous system: where the good guys go bad. Semin Cell Dev Biol.

[CR2] Ahonen M, Poukkula M, Baker AH, Kashiwagi M, Nagase H, Eriksson JE, Kähäri V-M (2003). Tissue inhibitor of metalloproteinases-3 induces apoptosis in melanoma cells by stabilization of death receptors. Oncogene.

[CR3] Blomstrand F, Giaume C (2006). Kinetics of endothelin-induced inhibition and glucose permeability of astrocyte gap junctions. J Neurosci Res.

[CR4] Blood CH, Sasse J, Brodt P, Zetter BR (1988). Identification of a tumor cell receptor for VGVAPG, an elastin-derived chemotactic peptide. J Cell Biol.

[CR5] Brassart B, Randoux A, Hornebeck W, Emonard H (1998). Regulation of matrix metalloproteinase-2 (gelatinase A, MMP-2), membrane-type matrix metalloproteinase-1 (MT1-MMP) and tissue inhibitor of metalloproteinases-2 (TIMP-2) expression by elastin-derived peptides in human HT-1080 fibrosarcoma cell line. Clin Exp Metastasis.

[CR6] Brew K, Dinakarpandian D, Nagase H (2000). Tissue inhibitors of metalloproteinases: evolution, structure and function. Biochim Biophys Acta Protein Struct Mol Enzymol.

[CR7] Cawston TE, Mercer E (1986). Preferential binding of collagenase to alpha 2-macroglobulin in the presence of the tissue inhibitor of metalloproteinases. FEBS Lett.

[CR8] Chang CH, Kawa Y, Tsai RK, Shieh JH, Lee JW, Watabe H, Kawakami T, Soma Y, Tajima S, Mizoguchi M (2008). Melanocyte precursors express elastin binding protein and elastin-derived peptide (VGVAPG) stimulates their melanogenesis and dendrite formation. J Dermatol Sci.

[CR9] Chen HH, Wang DL (2004) Nitric oxide inhibits matrix metalloproteinase-2 expression via the induction of activating transcription factor 3 in endothelial cells. Mol Pharmacol 65:1130–1140. https://doi.org/10.1124/mol.65.5.1130\r65/5/1130 10.1124/mol.65.5.1130 15102941

[CR10] Coquerel B, Poyer F, Torossian F, Dulong V, Bellon G, Dubus I, Reber A, Vannier JP (2009). Elastin-derived peptides: matrikines critical for glioblastoma cell aggressiveness in a 3-D system. Glia.

[CR11] Crocker SJ, Pagenstecher A, Campbell IL (2004). The TIMPs tango with MMPs and more in the central nervous system. J Neurosci Res.

[CR12] Cunningham LA, Wetzel M, Rosenberg GA (2005). Multiple roles for MMPs and TIMPs in cerebral ischemia. Glia.

[CR13] Devy J, Duca L, Cantarelli B, Joseph-Pietras D, Scandolera A, Rusciani A, Parent L, Thevenard J, Pasco SB, Tarpin M, Martiny L, Debelle L (2010). Elastin-derived peptides enhance melanoma growth in vivo by upregulating the activation of Mcol-A (MMP-1) collagenase. Br J Cancer.

[CR14] Docherty AJP, Lyons A, Smith BJ, Wright EM, Stephens PE, Harris TJR, Murphy G, Reynolds JJ (1985). Sequence of human tissue inhibitor of metalloproteinases and its identity to erythroid-potentiating activity. Nature.

[CR15] Donet M, Brassart-Pasco S, Salesse S, Maquart F-X, Brassart B (2014). Elastin peptides regulate HT-1080 fibrosarcoma cell migration and invasion through an Hsp90-dependent mechanism. Br J Cancer.

[CR16] Fager N, Jaworski DM (2000). Differential spatial distribution and temporal regulation of tissue inhibitor of metalloproteinase mRNA expression during rat central nervous system development. Mech Dev.

[CR17] Floquet N, Héry-Huynh S, Dauchez M, Derreumaux P, Tamburro AM, Alix AJP (2004). Structural characterization of VGVAPG, an elastin-derived peptide. Biopolymers.

[CR18] Gardner J, Ghorpade A (2003). Tissue inhibitor of metalloproteinase (TIMP)-1: the TIMPed balance of matrix metalloproteinases in the central nervous system. J Neurosci Res.

[CR19] Gmiński J, Mykała-Cieśla J, Machalski M, Dróżdż M (1992). Anti-elastin antibodies in patients with lung cancer. Immunol Lett.

[CR20] Gmiński J, Mykała-Cieśla J, Machalski M, Dróżdż M (1993). Elastin metabolism parameters in sera of patients with lung cancer. Neoplasma.

[CR21] Gomez DE, Alonso DF, Yoshiji H, Thorgeirsson UP (1997). Tissue inhibitors of metalloproteinases: structure, regulation and biological functions. Eur J Cell Biol.

[CR22] Groft LL, Muzik H, Rewcastle NB, Johnston RN, Knäuper V, Lafleur MA, Forsyth PA, Edwards DR (2001). Differential expression and localization of TIMP-1 and TIMP-4 in human gliomas. Br J Cancer.

[CR23] Guedez L, Courtemanch L, Stetler-Stevenson M (1998). Tissue inhibitor of metalloproteinase (TIMP)-1 induces differentiation and an antiapoptotic phenotype in germinal center B cells. Blood.

[CR24] Guedez L, Stetler-Stevenson WG, Wolff L, Wang J, Fukushima P, Mansoor A, Stetler-Stevenson M (1998). In vitro suppression of programmed cell death of B cells by tissue inhibitor of metalloproteinases-1. J Clin Invest.

[CR25] Hinek A, Rabinovitch M, Keeley F, Okamura-Oho Y, Callahan J (1993). The 67-kD elastin/laminin-binding protein is related to an enzymatically inactive, alternatively spliced form of beta-galactosidase. J Clin Invest.

[CR26] Jaworski DM, Fager N (2000). Regulation of tissue inhibitor of metalloproteinase-3 (Timp-3) mRNA expression during rat CNS development. J Neurosci Res.

[CR27] Jin R, Yang G, Li G (2010). Inflammatory mechanisms in ischemic stroke: role of inflammatory cells. J Leukoc Biol.

[CR28] Jung S, Rutka JT, Hinek A (1998). Tropoelastin and elastin degradation products promote proliferation of human astrocytoma cell lines. J Neuropathol Exp Neurol.

[CR29] Kettlun AM, Cartier L, García L, Collados L, Vásquez F, Ramírez E, Valenzuela MA (2003). TIMPs and MMPs expression in CSF from patients with TSP/HAM. Life Sci.

[CR30] Koh JY, Choi DW (1987). Quantitative determination of glutamate mediated cortical neuronal injury in cell culture by lactate dehydrogenase efflux assay. J Neurosci Methods.

[CR31] Koskivirta I, Rahkonen O, Mäyränpää M, Pakkanen S, Husheem M, Sainio A, Hakovirta H, Laine J, Jokinen E, Vuorio E, Kovanen P, Järveläinen H (2006). Tissue inhibitor of metalloproteinases 4 (TIMP4) is involved in inflammatory processes of human cardiovascular pathology. Histochem Cell Biol.

[CR32] Kurzepa J, Kurzepa J, Golab P, Czerska S, Bielewicz J (2014). The significance of matrix metalloproteinase (MMP)-2 and MMP-9 in the ischemic stroke. Int J Neurosci.

[CR33] Lipka D, Boratyński J (2008). Metaloproteinazy MMP. Struktura i funkcja Postepy Hig Med Dosw.

[CR34] Lombard C, Arzel L, Bouchu D, Wallach J, Saulnier J (2006). Human leukocyte elastase hydrolysis of peptides derived from human elastin exon 24. Biochimie.

[CR35] Ma R, Yuan B, Du J, Wang L, Ma L, Liu S, Shu Q, Sun H (2016). Electroacupuncture alleviates nerve injury after cerebra ischemia in rats through inhibiting cell apoptosis and changing the balance of MMP-9/TIMP-1 expression. Neurosci Lett.

[CR36] Maquart FX, Pasco S, Ramont L, Hornebeck W, Monboisse JC (2004). An introduction to matrikines: extracellular matrix-derived peptides which regulate cell activity - implication in tumor invasion. Crit Rev Oncol Hematol.

[CR37] Mitsiades N, Poulaki V, Kotoula V, Leone A, Tsokos M (1998). Fas ligand is present in tumors of the Ewing’s sarcoma family and is cleaved into a soluble form by a metalloproteinase. Am J Pathol.

[CR38] Moore CS, Milner R, Nishiyama A, Frausto RF, Serwanski DR, Pagarigan RR, Whitton JL, Miller RH, Crocker SJ (2011). Astrocytic tissue inhibitor of metalloproteinase-1 (TIMP-1) promotes oligodendrocyte differentiation and enhances CNS myelination. J Neurosci.

[CR39] Nicholson DW, Ali A, Thornberry NA, Vaillancourt JP, Ding CK, Gallant M, Gareau Y, Griffin PR, Labelle M, Lazebnik YA (1995). Identification and inhibition of the ICE/CED-3 protease necessary for mammalian apoptosis. Nature.

[CR40] Nicoloff G, Tzvetanov P, Christova P, Baydanoff S (2008). Detection of elastin derived peptides in cerebrospinal fluid of patients with first ever ischaemic stroke. Neuropeptides.

[CR41] Ntayi C, Labrousse AL, Debret R, Birembaut P, Bellon G, Antonicelli F, Hornebeck W, Bernard P (2004). Elastin-derived peptides upregulate matrix metalloproteinase-2-ediated melanoma cell invasion through elastin-binding protein. J Investig Dermatol.

[CR42] O’Rourke MF (2007). Arterial aging: pathophysiological principles. Vasc Med.

[CR43] Oh LY, Larsen PH, Krekoski CA, Edwards DR, Donovan F, Werb Z, Yong VW (1999). Matrix metalloproteinase-9/gelatinase B is required for process outgrowth by oligodendrocytes. J Neurosci.

[CR44] Phillips PG, Birnby LM (2004). Nitric oxide modulates caveolin-1 and matrix metalloproteinase-9 expression and distribution at the endothelial cell/tumor cell interface. Am J Physiol Lung Cell Mol Phys.

[CR45] Pocza P, Süli-Vargha H, Darvas Z, Falus A (2008). Locally generated VGVAPG and VAPG elastin-derived peptides amplify melanoma invasion via the galectin-3 receptor. Int J Cancer.

[CR46] Robinet A (2005). Elastin-derived peptides enhance angiogenesis by promoting endothelial cell migration and tubulogenesis through upregulation of MT1-MMP. J Cell Sci.

[CR47] Saura J (2007). Microglial cells in astroglial cultures: a cautionary note. J Neuroinflammation.

[CR48] Senior RM, Griffin GL, Mecham RP, Wrenn DS, Prasad KU, Urry DW (1984). Val-Gly-Val-Ala-Pro-Gly, a repeating peptide in elastin, is chemotactic for fibroblasts and monocytes. J Cell Biol.

[CR49] Seo D-W, Li H, Guedez L, Wingfield PT, Diaz T, Salloum R, Wei B, Stetler-Stevenson WG (2003). TIMP-2 mediated inhibition of angiogenesis. Cell.

[CR50] Shin CY, Lee WJ, Choi JW, Choi MS, Ryu JR, Oh SJ, Cheong JH, Choi EY, Ko KH (2007). Down-regulation of matrix metalloproteinase-9 expression by nitric oxide in lipopolysaccharide-stimulated rat primary astrocytes. Nitric Oxide.

[CR51] Siemianowicz K, Gminski J, Goss M, Francuz T, Likus W, Jurczak T, Garczorz W (2010). Influence of elastin-derived peptides on metalloprotease production in endothelial cells. Exp Ther Med.

[CR52] Skeie JM, Hernandez J, Hinek A, Mullins RF (2012). Molecular responses of choroidal endothelial cells to elastin derived peptides through the elastin-binding protein (GLB1). Matrix Biol.

[CR53] Stetler-Stevenson WG, Bersch N, Golde DW (1992). Tissue inhibitor of metalloproteinase-2 (TIMP-2) has erythroid-potentiating activity. FEBS Lett.

[CR54] Szychowski KA, Sitarz AM, Wojtowicz AK (2015). Triclosan induces Fas receptor-dependent apoptosis in mouse neocortical neurons in vitro. Neuroscience.

[CR55] Szychowski KA, Leja ML, Kaminskyy DV, Kryshchyshyn AP, Binduga UE, Pinyazhko OR, Lesyk RB, Tobiasz J, Gmiński J (2017). Anticancer properties of 4-thiazolidinone derivatives depend on peroxisome proliferator-activated receptor gamma (PPARγ). Eur J Med Chem.

[CR56] Trojanek JB (2015). Role of matrix metalloproteinases and tissue inhibitors of metalloproteinases in hypertension. Pathogenesis of hypertension and obesity. Postepy Biochem.

[CR57] Tzvetanov P, Nicoloff G, Rousseff R, Christova P (2008). Increased levels of elastin-derived peptides in cerebrospinal fluid of patients with lacunar stroke. Clin Neurol Neurosurg.

[CR58] Vaalamo M, Leivo T, Saarialho-Kere U (1999). Differential expression of tissue inhibitors of metalloproteinases (TIMP-1, -2, -3, and -4) in normal and aberrant wound healing. Hum Pathol.

[CR59] Vaillant C, Didier-Bazès M, Hutter A, Belin MF, Thomasset N (1999). Spatiotemporal expression patterns of metalloproteinases and their inhibitors in the postnatal developing rat cerebellum. J Neurosci.

[CR60] Vanmeter TE, Rooprai HK, Kibble MM, Fillmore HL, Broaddus WC, Pilkington GJ (2001). The role of matrix metalloproteinase genes in glioma invasion: co-dependent and interactive proteolysis. J Neuro-Oncol.

[CR61] Vitvitsky V, Thomas M, Ghorpade A, Gendelman HE, Banerjee R (2006). A functional transsulfuration pathway in the brain links to glutathione homeostasis. J Biol Chem.

[CR62] Wang JY, Shum AY, Hwang CP (1998). Ethanol modulates induction of nitric oxide synthase in glial cells by endotoxin. Life Sci.

[CR63] Wang XX, Tan MS, Yu JT, Tan L (2014). Matrix metalloproteinases and their multiple roles in Alzheimer’s disease. Biomed Res Int.

[CR64] Yong VW, Power C, Forsyth P, Edwards DR (2001). Metalloproteinases in biology and pathology of the nervous system. Nat Rev Neurosci.

