# Supplementary material for: Impact of Elastin-Derived Peptide VGVAPG on Matrix Metalloprotease-2 and -9 and the Tissue Inhibitor of Metalloproteinase-1, -2, -3 and -4 mRNA Expression in Mouse Cortical Glial Cells In Vitro
Source: Neurotox Res. 2018 Jul 30;35(1):100–10. doi: 10.1007/s12640-018-9935-x (PMC6313372; doi:10.1007/s12640-018-9935-x)
Supplement: Supplementary file 1 — (PPTX 4022 kb) [file 12640_2018_9935_MOESM1_ESM.pptx]

## Slide 1
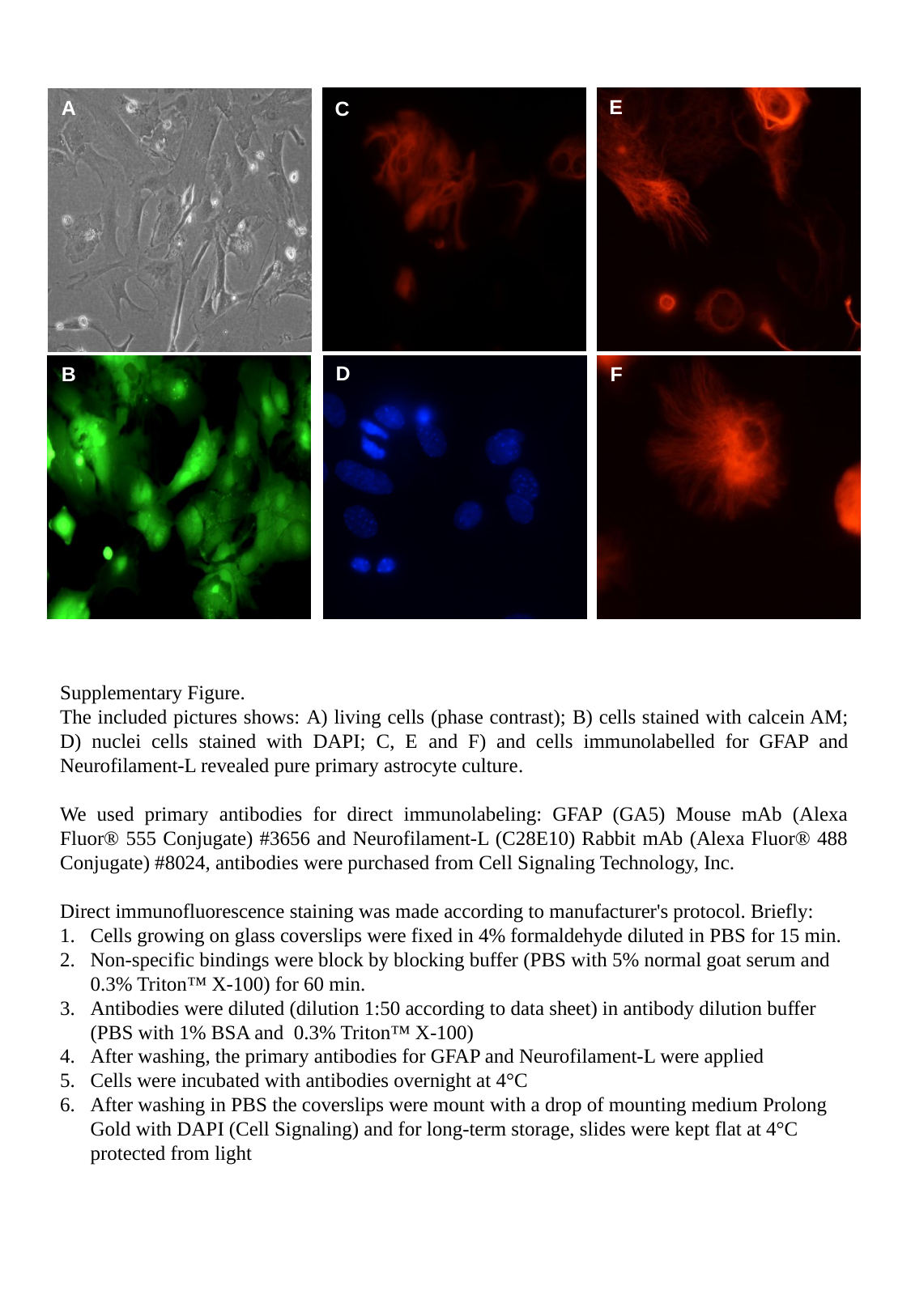

E
A
C
D
B
F
Supplementary Figure.
The included pictures shows: A) living cells (phase contrast); B) cells stained with calcein AM; D) nuclei cells stained with DAPI; C, E and F) and cells immunolabelled for GFAP and Neurofilament-L revealed pure primary astrocyte culture.
We used primary antibodies for direct immunolabeling: GFAP (GA5) Mouse mAb (Alexa Fluor® 555 Conjugate) #3656 and Neurofilament-L (C28E10) Rabbit mAb (Alexa Fluor® 488 Conjugate) #8024, antibodies were purchased from Cell Signaling Technology, Inc.
Direct immunofluorescence staining was made according to manufacturer's protocol. Briefly:
Cells growing on glass coverslips were fixed in 4% formaldehyde diluted in PBS for 15 min.
Non-specific bindings were block by blocking buffer (PBS with 5% normal goat serum and 0.3% Triton™ X-100) for 60 min.
Antibodies were diluted (dilution 1:50 according to data sheet) in antibody dilution buffer (PBS with 1% BSA and 0.3% Triton™ X-100)
After washing, the primary antibodies for GFAP and Neurofilament-L were applied
Cells were incubated with antibodies overnight at 4°C
After washing in PBS the coverslips were mount with a drop of mounting medium Prolong Gold with DAPI (Cell Signaling) and for long-term storage, slides were kept flat at 4°C protected from light
